# Identification of stable reference genes for quantitative gene expression analysis in the duodenum of meat-type ducks

**DOI:** 10.3389/fvets.2023.1160384

**Published:** 2023-04-03

**Authors:** Fei Shui, Guiru Qiu, Shenqiang Pan, Xin Wang, Fumin Jia, Tingting Jiang, Yongsheng Li, Zhaoyu Geng, Sihua Jin

**Affiliations:** ^1^College of Animal Science and Technology, Anhui Agricultural University, Hefei, China; ^2^Anhui Provincial Key Laboratory of Local Animal Genetic Resources Conservation and Bio-Breeding, Hefei, China; ^3^Extension Center for Animal Husbandry and Veterinary Medicine of Huangshan City, Huangshan, China

**Keywords:** duodenal epithelial, gene expression, meat-type ducks, reference genes, qPCR

## Abstract

Quantitative polymerase chain reaction (qPCR) is an important method to detect gene expression at the molecular level. The selection of appropriate housekeeping genes is the key to accurately calculating the expression level of target genes and conducting gene function studies. In this study, the expression of eight candidate reference genes, glyceraldehyde-3-phosphate dehydrogenase (*GAPDH*), beta-actin (β*-actin*), 18S ribosomal RNA (*18S rRNA*), hydroxymethylbilane synthase (*HMBS*), hypoxanthine phosphoribosyltransferase 1 (*HPRT1*), TATA box binding protein (*TBP*), ribosomal protein L13 (*RPL13*), and tyrosine 3-monooxygenase/tryptophan 5-monooxygenase activation protein (*YWHAZ*), in the duodenal epithelial tissue of 42-day-old meat-type ducks were detected using qPCR. Furthermore, their expression stability was analyzed using the geNorm, NormFinder, and BestKeeper programs. The results indicated that *HMBS* and *YWHAZ* were the most stably expressed genes. All three programs indicated that the expression of *18S rRNA* was the least stable, making it unsuitable for the study of gene expression in meat-type duck tissues. This study provides stable reference genes for gene expression analysis and contributes to further studies on the gene function of meat-type ducks.

## Introduction

Reference genes, often referred to as housekeeping genes, are typically used as a reference to normalize mRNA levels between different samples since the expression levels of genes may vary across tissues or cells in some cases (1, 2). Quantitative polymerase chain reaction (qPCR) is an important tool in molecular biology research. It has the advantages of high specificity, high sensitivity, a high degree of automation, and accurate quantification (3). Nowadays, qPCR has become a common method to study the genetic mechanisms of some growth traits that are beneficial to human beings and livestock. Reference genes have an important influence on the process of qPCR because the standardization of these genes controls the accuracy of qPCR (4). In scientific research, the best internal reference gene should have a relatively stable expression level in different stages of development and different tissues of organisms. However, many experiments have shown that no single gene can reach a constant level of expression under such different conditions (5–7).

A total of eight commonly used reference genes were selected, based on existing studies. Godornes et al. (8) reported that the hypoxanthine phosphoribosyltransferase 1 (*HPRT1*) gene is a stable reference gene in rabbits. Another study on chickens found that the most suitable reference gene in the liver is ribosomal protein L13 (*RPL13*); in the jejunum it is glyceraldehyde-3-phosphate dehydrogenase (*GAPDH*), while hydroxymethylbilane synthase (*HMBS*) is compatible in all tissues (9). In pigs, *HPRT1* and *HMBS* are reference genes for skeletal muscles and affect their postnatal growth (10). In goats, beta-actin (β*-actin*) was the most stable in the stomach, small intestine, and ovary; 18S ribosomal RNA (*18S rRNA*) in the heart and spleen; *HMBS* in the uterus and lungs; TATA box binding protein (*TBP*) in the liver; *HPRT1* in the kidney; and *GAPDH* in the muscles (11). While all the above genes can be employed as stable reference genes for rabbits, pigs, and other model animals, there is a lack of stable reference genes for conducting gene expression studies in meat-type ducks. Filling this gap in research has been made more pertinent by the growth of the meat-type duck industry in China, becoming an important livestock and poultry industry in the country's rural economic development.

This study, therefore, considered meat-type duck as the research subject and, based on qPCR technology and comprehensive use of the geNorm, NormFinder, and BestKeeper programs, assessed the stability of multiple candidate reference genes, aiming to identify a list of effective reference genes for quantitative gene expression analysis in meat-type ducks, which will be beneficial to further gene function studies.

## Materials and methods

### Ethics statement

All the study procedures that included animals were strictly carried out under the regulations and guidelines established by the Administration of Affairs Concerning Experimental Animals (Ministry of Science and Technology, China, revised in June 2004). All experimental procedures were reviewed and approved by the Institutional Animal Care and Use Committee of Anhui Agricultural University (SYXK 2016-007). The animals in the study had *ad libitum* access to water and feed (Table 1) and were humanely sacrificed.

**Table 1:** Nutrient and energy content of the feeds.

**Item**	**0–21 d**	**22–42 d**
Metabolizable energy, kcal/kg	2,900	2,940
Crude protein (%)	20.03	17.50
Methionine (%)	0.44	0.40
Cystine (%)	0.37	0.32
Lysine (%)	1.08	0.91
Calcium (%)	0.90	0.86
Non-phytate phosphorus (%)	0.40	0.38

### Sample preparation

A total of sixteen 42-day-old male meat-type ducks were provided by Huangshan Qiangying Duck Breeding Co. Ltd. (Anhui, China). At 21 days, all ducks were reared in the individual cages (55 × 50 × 40 cm) for feeding until 42 days of age. All ducks were exposed to continuous illumination (24 L:0 D) for the first 72 h after hatching, followed by a 20 L:4 D lighting regime until the end of the experiment. All ducks were kept in the same house and fed the same basal diet at room temperature. After slaughtering, duodenal tissues of the meat-type ducks were collected and immediately placed in a 2.0 mL centrifuge tube with RNALater at 4°C overnight and stored at −80°C for further use.

The total RNA from duodenal epithelial tissues was extracted using the total RNA Kit (Omega Bio-Tek, Doraville, GA, USA) following the manufacturer's instructions. The RNA concentration and purity of samples were determined by the NanoDrop spectrophotometer (Thermo Fisher Scientific, New York, NY, USA) and 1.0% agarose gel electrophoresis. SuperMix (Yeasen, Shanghai, China) was used to reverse the transcription of the isolated total RNA into cDNA; then, qPCR was performed. All experimental procedures were performed as per the manufacturer's protocol and conducted on a sterilized bench in a clean room.

### Quantitative PCR

According to the duck gene sequence published in GenBank, Table 2 shows that the primers of eight candidate reference genes were designed using Primer Premier 5.0 software and UCSC *In-Silico* PCR were used to test the specificity and sensitivity (http://genome.ucsc.edu/). The cDNA obtained in the previous step served as a template; the reference genes *GAPDH*, β*-actin, 18S rRNA, HMBS, HPRT1, TBP, RPL13*, and tyrosine 3-monooxygenase/tryptophan 5-monooxygenase activation protein (*YWHAZ*) were used for conventional PCR amplification; and the primer specificity and amplification efficiency were detected by gel electrophoresis and qPCR. PCR was implemented in a 20.0 μL reaction containing 8.2 μL ddH_2_O, 10.0 μL SYBR Green Master Mix (Yeasen, Shanghai, China), 1.0 μL template DNA, and 0.4 μL of each primer. The reaction conditions were 95°C for 5 min; 95°C for 10 s, 60°C for 20 s, 72°C for 20 s, 40 cycles; 95°C for 15 s, 60°C for 1 min, 95°C for 15 s. The sample copy number of logarithmic scales was taken as the abscissa and the Ct value as the ordinate, and the testing purpose gene amplification curve, melting curve, and standard curve were obtained. The qualified primers could then be used for subsequent experiments.

**Table 2:** Primers used for quantitative PCR.

**Gene^1^**	**Accession no**.	**Primer^2^ (5^′^-3^′^)**	**Length (bp)**	**Annealing temperature (°C)**
*GAPDH*	XM_027449740	F: TGCCATCACAGCCACACAGA	166	60
		R: ACACCCAAGGAAGGCCAACA		
*β-actin*	EF667345	F: GATGAATCCGGACCCTCCAT	71	60
		R: AAGGGTGTGGGTGTTGGTAA		
*18S rRNA*	XR_003493879	F: GAGCCTGCGGCTTAATTTGA	121	60
		R: AACTAAGAACGGCCATGCAC		
*HMBS*	XM_027444343	F: TGGACCAAATGACGATGTGC	174	60
		R: CCACAGGTTTAGCAGGCATC		
*HPRT1*	XM_027465333	F: GCTTGAACGCTGGCAAGATA	68	60
		R: TGCAGTCTCAGTCCTGTTGT		
*TBP*	XM_027454201	F: TTAGCCCGATGATGCCGTAT	198	60
		R: GGGCTGTGGTAAGAGTCTGT		
*RPL13*	XM_027466423	F: GCATGATCCTGAAGCCGCAC	166	60
		R: TGGTCGGACACCTCACGATG		
*YWHAZ*	XM_027452537	F: TGCTGCTGGAGATGACAAGA	106	60
		R: TCTGATGGGATGTGTTGGCT		

^1^*GAPDH*, Glyceraldehyde-3-phosphate dehydrogenase; β*-actin*, beta-actin; *18S rRNA*, 18S ribosomal RNA; *HMBS*, hydroxymethylbilane synthase; *HPRT1*, hypoxanthine phosphoribosyltransferase 1; *TBP*, TATA box binding protein; *RPL13*, ribosomal protein L13; *YWHAZ*, Tyrosine 3-monooxygenase/tryptophan 5-monooxygenase activation protein zeta polypeptide.

^2^F, forward; R reverse.

### Evaluation of stable reference gene expression and statistical analyses

Based on the mRNA expression of reference genes, three types of stability evaluation software, geNorm, NormFinder, and BestKeeper, were used for statistical analysis to select the stable reference genes. The test data obtained from qPCR of the reference genes were exported to Microsoft Excel 2019 (Microsoft, Redmond, WA, USA). All three software packages were used as per the manufacturer's instructions.

geNorm is a program designed by Vandesompele et al. (4) for selecting the most stable reference genes and determining the optimal reference genes in qPCR. geNorm achieves the rank of internal reference genes by calculating the average expression stability. Each dataset was transformed to relative quantities using the 2^−Δ*Ct*^ method (ΔCt = the corresponding Ct value – minimum Ct value). NormFinder (https://moma.dk/normfinder-software) is a program designed by Andersen et al. (12) to screen stable reference genes. The computational mechanism of this program is the same as that of the geNorm program. NormFinder also uses the 2^−Δ*Ct*^ method, derives relative quantities from raw Ct values, and then calculates and ranks each dataset. BestKeeper (https://www.gene-quantification.de/bestkeeper.html) is a program written by Pfaffl et al. (13) for the expression analysis of housekeeping and target genes. BestKeeper can directly calculate the stability using the Ct value of gene expression. The Ct value of each sample gene was entered into an Excel spreadsheet, and then BestKeeper internal stability analysis software was imported for calculation. Through the input of Ct, the program calculated the correlation coefficient (*r*), standard deviation (SD), and coefficient of variation (CV) for each gene to generate the pairing and then compared the size of each value to determine the reference gene with good stability. The larger the *R*, the smaller the SD and CV, and the better the stability of the reference gene. SD > 1 indicated that the expression of a housekeeping gene was not stable. Finally, by averaging the rank of the eight reference genes obtained using the three software, the stability of the genes was comprehensively evaluated, and the most suitable housekeeping genes were derived.

## Results

### RNA isolation and cDNA synthesis

Total RNA samples were analyzed using 1.0% agarose gel electrophoresis (Supplementary Figure 1). Two bands (representing 28 S and 18 S) were detected; the 28 S band was brighter than the 18 S band. The OD260/OD280 ratios of the samples' RNA were all 1.9–2.1, which showed that the isolated total RNA was of sufficient purity and without degradation.

### Expression analysis of housekeeping genes

**Figure 1:**
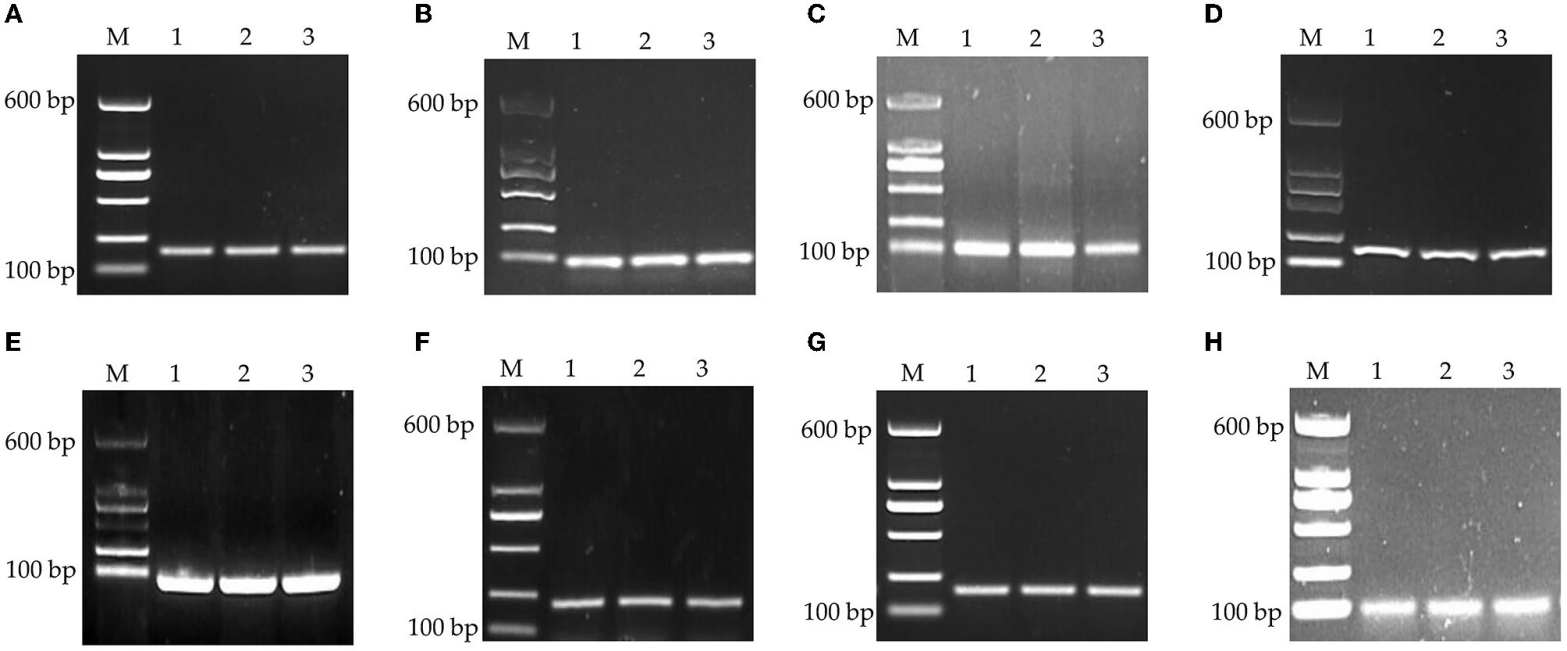
PCR products of housekeeping genes by agarose gel electrophoresis. **(A)** *GAPDH*, **(B)** β*-actin*, **(C)** *18S rRNA*, **(D)** *HMBS*, **(E)** *HPRT1*, **(F)** *TBP*, **(G)** *RPL13*, and **(H)** *YWHAZ*. M: DNA marker.

The specificity was assessed by visualization of specific amplified product sizes by electrophoresis in agarose gel (1.5%). As shown in Figure 1, the results of the amplification revealed that the fragment lengths of the products of *GAPDH*, β*-actin, 18S rRNA, HMBS, HPRT1, TBP, RPL13*, and *YWHAZ* were consistent with their theoretical lengths. This indicated that the cDNA generated by reverse transcription had good integrity and amplification product specificity and could be used in the follow-up experiment.

### Stability of reference genes by geNorm

geNorm analyzed the expression stability of the eight candidate housekeeping genes in the duodenum of meat-type ducks. As per the principle that the smaller the average stability value (M), the more stable the gene expression, the ranking of the eight housekeeping genes is represented in Table 3. Using the transformed data, geNorm generated a graph considering the stability value in which low stability values characterized genes with the most stable expression. The stability rank of each housekeeping gene was ranked as follows: *HMBS* = *YWHAZ* > β*-actin* > *RPL13* > *GAPDH* > *TBP* > *HPRT1* > *18S rRNA*. The results showed that the most stable internal genes were *HMBS* and *YWHAZ*, while the least stable was *18S rRNA*.

**Table 3:** Average expression stability values of candidate reference genes calculated by geNorm.

**Gene**	**Average stability value (M)**	**Rank**
*HMBS*	0.337	1
*YWHAZ*	0.337	1
*β-actin*	0.511	2
*RPL13*	0.706	3
*GAPDH*	0.777	4
*TBP*	0.846	5
*HPRT1*	1.003	6
*18S rRNA*	1.150	7

*GAPDH*, Glyceraldehyde-3-phosphate dehydrogenase; β*-actin*, beta-actin; *18S rRNA*, 18S ribosomal RNA; *HMBS*, hydroxymethylbilane synthase; *HPRT1*, hypoxanthine phosphoribosyltransferase 1; *TBP*, TATA box binding protein; *RPL13*, ribosomal protein L13; *YWHAZ*, Tyrosine 3-monooxygenase/tryptophan 5-monooxygenase activation protein zeta polypeptide.

### Stability of reference genes by NormFinder

NormFinder calculated the stable values of the eight reference genes in the duodenal epithelial cells of meat-type ducks. The smaller the stability value (S) of a gene, the more stable it is. The stability of each reference gene was ranked as follows (Table 4): *HMBS* > *YWHAZ* > β*-actin* > *RPL13* > *GAPDH* > *TBP* > *HPRT1* > *18S rRNA*. The results revealed that the most stable internal gene was *HMBS*, while the least stable was *18S rRNA*.

**Table 4:** Average expression stability values of candidate reference genes calculated by NormFinder.

**Gene**	**Stability value (S)**	**Rank**
*HMBS*	0.052	1
*YWHAZ*	0.117	2
*β-actin*	0.462	3
*RPL13*	0.527	4
*GAPDH*	0.556	5
*TBP*	0.579	6
*HPRT1*	0.858	7
*18S rRNA*	0.987	8

*GAPDH*, Glyceraldehyde-3-phosphate dehydrogenase; β*-actin*, beta-actin; *18S rRNA*, 18S ribosomal RNA; *HMBS*, hydroxymethylbilane synthase; *HPRT1*, hypoxanthine phosphoribosyltransferase 1; *TBP*, TATA box binding protein; *RPL13*, ribosomal protein L13; *YWHAZ*, Tyrosine 3-monooxygenase/tryptophan 5-monooxygenase activation protein zeta polypeptide.

### Stability of reference genes by BestKeeper

The determination of reference gene stability by BestKeeper program analysis was done by directly inputting the Ct value of each gene into an Excel table and importing it into the BestKeeper program. The stability rank of each reference gene by BestKeeper was as follows (Table 5): *HMBS* = *YWHAZ* > β*-actin* > *RPL13* > *HPRT1* > *GAPDH* > *TBP* > *18S rRNA*. The results indicated that *HMBS* and *YWHAZ* were the most stably expressed genes; *18S rRNA* was the least stably expressed.

**Table 5:** Average expression stability values of candidate reference genes calculated by BestKeeper.

**Gene**	**Standard deviation (SD)**	**Coefficient of variation (CV)**	**Correlation of coefficient (r)**	***p***	**Rank**
*HMBS*	0.001	2.76	0.654	0.001	1
*YWHAZ*	0.001	2.54	0.862	0.001	1
*β-actin*	0.007	4.82	0.036	0.898	2
*RPL13*	0.015	2.68	0.438	0.090	3
*HPRT1*	0.043	3.12	0.505	0.046	4
*GAPDH*	0.102	2.21	0.868	0.001	5
*TBP*	0.254	4.04	0.655	0.006	6
*18S rRNA*	0.754	3.48	0.579	0.019	7

*GAPDH*, Glyceraldehyde-3-phosphate dehydrogenase; β*-actin*, beta-actin; *18S rRNA*, 18S ribosomal RNA; *HMBS*, hydroxymethylbilane synthase; *HPRT1*, hypoxanthine phosphoribosyltransferase 1; *TBP*, TATA box binding protein; *RPL13*, ribosomal protein L13; *YWHAZ*, Tyrosine 3-monooxygenase/tryptophan 5-monooxygenase activation protein zeta polypeptide.

### Comprehensive evaluation of reference genes' expression stability

**Figure 2:**
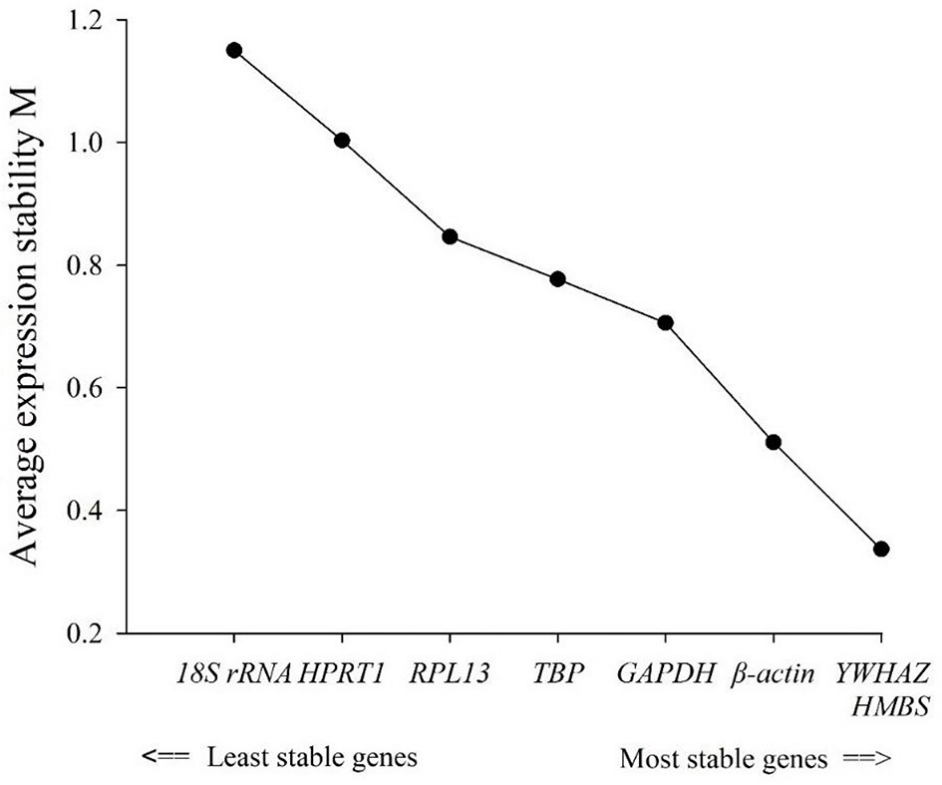
Average expression stability (M).

The comprehensive evaluation results of the eight housekeeping genes selected by geNorm, NormFinder, and BestKeeper are shown in Table 6 and Figure 2. From geNorm, *HMBS* and *YWHAZ* were determined to be the most stable genes in duodenal epithelial cells, followed by β*-actin, RPL13, GAPDH, TBP, HPRT1*, and *18S rRNA*. From NormFinder, *HMBS* was identified to be the most stably expressed reference gene, followed by *YWHAZ*, β*-actin, RPL13, GAPDH, TBP, HPRT1*, and *18S rRNA*. BestKeeper revealed that *HMBS* and *YWHAZ* were the most stable genes, followed by β*-actin, RPL13, HPRT1, GAPDH, TBP*, and *18S rRNA*. The comprehensive analysis results showed that *HMBS* and *YWHAZ* are relatively the most stable reference genes in the duodenal epithelial cells of meat-type ducks, followed by β*-actin*, while *18S rRNA, HPRT1*, and *TBP* are the least stable. Therefore, *HMBS* and *YWHAZ* can be selected as the best reference genes in the duodenal epithelial cells of meat-type ducks.

**Table 6:** Integrated table of reference gene expression stability values by the three different statistical methods.

**Gene**	**Program**			**Mean rank**
	**geNorm**	**NormFinder**	**BestKeeper**	
*HMBS*	1	1	1	1.00
*YWHAZ*	1	2	1	1.33
*β-actin*	2	3	2	2.33
*RPL13*	3	4	3	3.33
*GAPDH*	4	5	5	4.67
*TBP*	5	6	6	5.67
*HPRT1*	6	7	4	5.67
*18S rRNA*	7	8	7	7.33

*GAPDH*, Glyceraldehyde-3-phosphate dehydrogenase; β*-actin*, beta-actin; *18S rRNA*, 18S ribosomal RNA; *HMBS*, hydroxymethylbilane synthase; *HPRT1*, hypoxanthine phosphoribosyltransferase 1; *TBP*, TATA box binding protein; *RPL13*, ribosomal protein L13; *YWHAZ*, Tyrosine 3-monooxygenase/tryptophan 5-monooxygenase activation protein zeta polypeptide.

## Discussion

In animal breeding research, gene expression is important for understanding biological processes. The qPCR technique has become widely used to detect gene expression functions (14). Many factors in qPCR, including the selection of housekeeping genes, may impact the results. To make the analysis of the expression of a particular gene more accurate, there must be a reliable way to calculate the expression. Therefore, a suitable internal reference gene is needed to reliably quantify gene transcripts. However, research pertaining to the suitable confirmation of the stability of expression levels when these housekeeping genes are used is scarce.

Housekeeping gene expression vary in different tissues and under different experimental conditions (15). For example, as the most used housekeeping gene, *GAPDH* was observed to be the most available internal reference gene for expression investigations in human reticulocytes (16–18). However, a study on virus-infected salmon reached the opposite conclusion (19, 20). We also observed that *GAPDH* was not the best internal reference gene in the duodenum of meat-type ducks. Therefore, different housekeeping genes should be identified for study in different animals or experiments.

Previous studies have shown that *HMBS* is the most stable housekeeping gene in human hepatocellular carcinoma tissue, blood samples (21), and brain tissue (22). Moreover, studies have found that *HMBS* has good stability in broilers (9, 23), layers (24), and quails (25). The above research results are consistent with the results of the experiments in this study, suggesting that *HMBS* may be the most suitable gene as an internal reference gene in birds. Another study mentioned that *HMBS* is highly specific to avian species (26). Based on this, *HMBS* can help reduce the possibility of false negative tests caused by insufficient sampling or storage degradation.

Moreover, Dai et al. (27) found that *YWHAZ* ranked high in the search for reference genes of fetal mice, which is suitable to be used in the study together with other reference genes. *YWHAZ* is considered the most stable reference gene in sheep leukocytes (28) and hypothalamic-pituitary-gonadal axis tissues of sows (29), which can serve for accurate and repeatable qPCR data analysis. Previous studies have shown that *YWHAZ* is a relatively suitable reference gene in multiple avian species, allowing for accurate normalization and quantification of gene expression levels in a variety of avian species (30–34).

In our study, we applied qPCR to detect the expression of eight candidate reference genes, *GAPDH*, β*-actin, 18S rRNA, HMBS, HPRT1, TBP, RPL13*, and *YWHAZ*, in the duodenal epithelial tissue of 42-day-old meat-type ducks and used the geNorm, NormFinder, and BestKeeper programs to investigate the expression stability. These software are commonly used to identify reference genes stably expressed (32, 35–37). The stability of each gene, as analyzed by different software programs, was inconsistent owing to different algorithms. geNorm prefers to compute the stability value of gene expression and estimate the number of housekeeping genes, whereas NormFinder focuses on the CV of a gene across all samples, and BestKeeper directly analyzes Ct values. Although some differences were observed in the final results owing to the distinct algorithms of the three programs, the ranking of the eight genes studied was the same. Considering the evaluation results of the three programs, *HMBS* and *YWHAZ* can be considered relatively stable internal reference genes in duodenal epithelial cells, and *18S rRNA, HPRT1*, and *TBP* relatively unstable. The results of this study yield a reference basis for the applicable normalization of housekeeping genes under diverse experimental conditions.

In summary, our results show that *HMBS* and *YWHAZ* are the most stable reference genes in meat-type ducks, while *18S rRNA* is the least stable and is not suitable for studying gene expression in meat-type duck tissues. Our study highlights the value of reference genes in the use of qPCR and lays the foundation for the application of quantitative gene expression analysis to elucidate gene function studies in modern poultry breeding programs.

## Data availability statement

The raw data supporting the conclusions of this article will be made available by the authors, without undue reservation.

## Ethics statement

All the study procedures that included animals were strictly carried out under the regulations and guidelines established by the Administration of Affairs Concerning Experimental Animals (Ministry of Science and Technology, China, revised in June 2004). All experimental procedures were reviewed and approved by the Institutional Animal Care and Use Committee of Anhui Agricultural University (SYXK 2016-007). The animals in the study had *ad libitum* access to water and feed (Table 1) and were humanely sacrificed.

## Author contributions

SJ and ZG conceived this study. FS, GQ, SP, XW, YL, TJ, and FJ collected the samples and performed the experiments. FS, SP, and SJ analyzed the data. FS, GQ, and SJ wrote the manuscript. SJ, FS, GQ, and ZG participated in the planning of experiments and revising the manuscript. All authors have read and approved the final version of the manuscript.

## Supplementary material

Supplementary Figure S1 The agarose electrophoresis of total RNA.
