# Supplementary figures and images for: Identification of stable reference genes for quantitative gene expression analysis in the duodenum of meat-type ducks
Source: Front Vet Sci. 2023 Apr 3;10:1160384. doi: 10.3389/fvets.2023.1160384 (PMC10106614; doi:10.3389/fvets.2023.1160384)

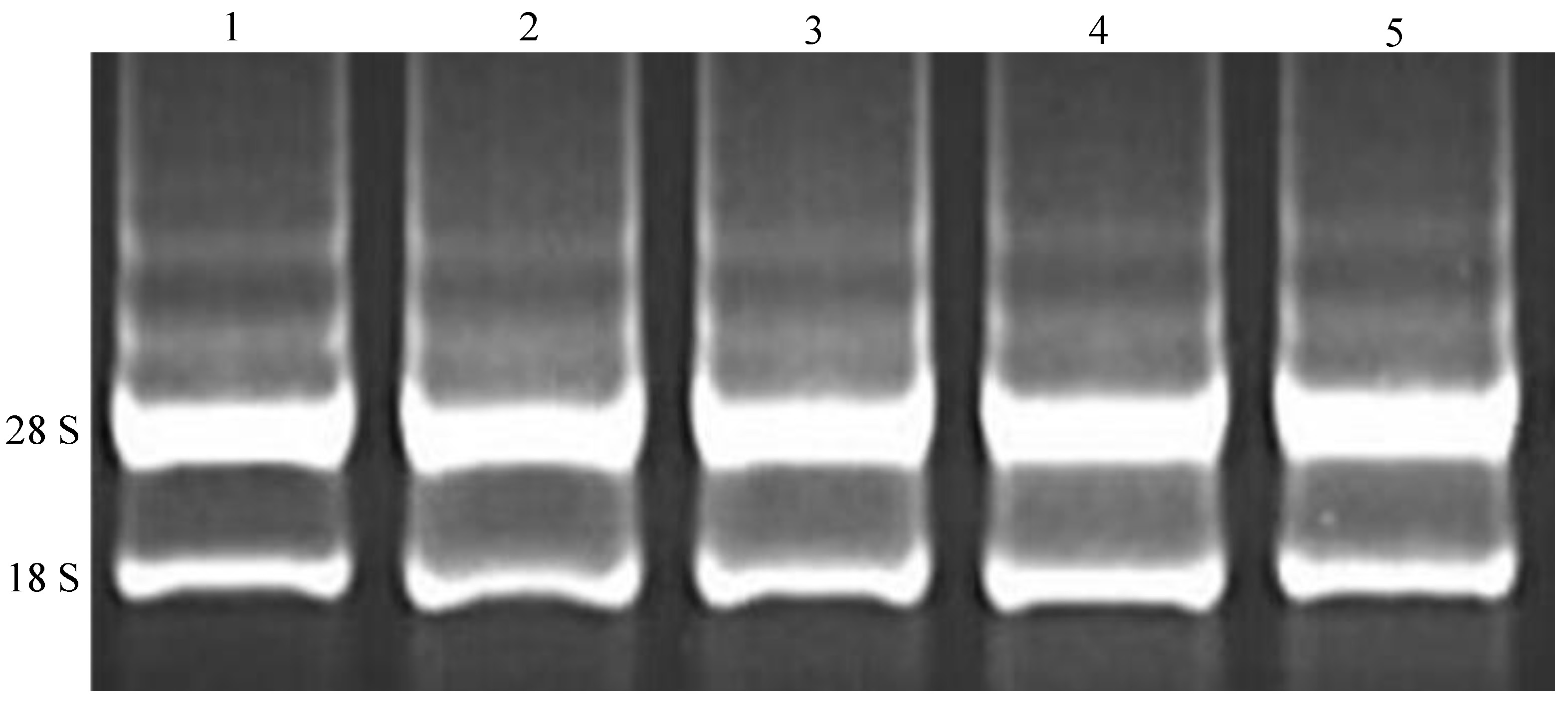

Supplement: Supplementary Figure S1 — The agarose electrophoresis of total RNA. [file Image_1.JPEG]
